# Efficacy and safety of semaglutide in patients with heart failure with preserved ejection fraction and obesity

**DOI:** 10.1002/clc.24283

**Published:** 2024-05-20

**Authors:** Ayesha Rehman, Shahab Saidullah, Muhammad Asad, Umer R. Gondal, Amna Ashraf, Muhammad F. Khan, Waheed Akhtar, Amin Mehmoodi, Jahanzeb Malik

**Affiliations:** ^1^ Department of Medicine Quaid e Azam Medical College Bahawalpur Pakistan; ^2^ Department of Cardiology Pakistan Institute of Medical Sciences Islamabad Pakistan; ^3^ Department of Cardiology Benazir Bhutto Hospital Rawalpindi Pakistan; ^4^ Department of Medicine Reading Hospital West Reading PA USA; ^5^ Department of Medicine Military Hospital Rawalpindi Pakistan; ^6^ Department of Medicine Ayub Teaching Hospital Abbottabad Pakistan; ^7^ Department of Cardiology Abbas Institute of Medical Sciences Muzaffrabad Pakistan; ^8^ Department of Medicine Ibn e Seena Hospital Kabul Afghanistan; ^9^ Department of Cardiovascular Medicine Cardiovascular Analytics Group Islamabad Pakistan

**Keywords:** exercise capacity, heart failure with preserved ejection fraction, obesity, semaglutide, weight management

## Abstract

**Background:**

Semaglutide, a once‐weekly glucagon‐like peptide‐1 receptor agonist, has shown promise in weight management and cardiovascular outcomes in other populations. This study aimed to evaluate the efficacy of semaglutide in heart failure with preserved ejection fraction (HFpEF) patients with obesity.

**Methods:**

A retrospective study analyzed 318 patients with HFpEF, of which 104 received semaglutide and 214 received placebo. Primary endpoints included evaluating changes in exercise capacity and weight management.

**Results:**

Semaglutide treatment led to significant improvements in the primary endpoints. Patients in the semaglutide group demonstrated substantial enhancements in exercise capacity, as measured by the 6‐min walk distance, compared to the placebo group (mean difference 15.1 meters, 95% CI 5.8 to 24.4, *p* = 0.002). Additionally, semaglutide resulted in substantial weight loss compared to placebo (mean difference −2.9%, 95% CI −4.1–−1.7, *p* = 0.001). Several secondary endpoints, including reductions in C‐reactive protein levels and improvements in other clinical parameters, further supported the efficacy of semaglutide. Adverse events were generally well‐tolerated, with no unexpected safety concerns.

**Conclusion:**

Semaglutide demonstrated significant clinical benefits in HFpEF patients with obesity, as evidenced by improved symptoms, physical function, and weight reduction.

## Graphical abstract

This retrospective study assessed semaglutide, a weekly glucagon‐like peptide‐1 receptor agonist, in 318 patients with heart failure with preserved ejection fraction and obesity. Semaglutide demonstrated significant improvements in exercise capacity and weight management compared to the placebo group. Secondary endpoints, including reductions in C‐reactive protein levels, further supported its clinical benefits. Adverse events were generally well‐tolerated, with no unexpected safety concerns. Overall, semaglutide showed promise in enhancing symptoms, physical function, and inducing weight reduction in HFpEF patients with obesity.

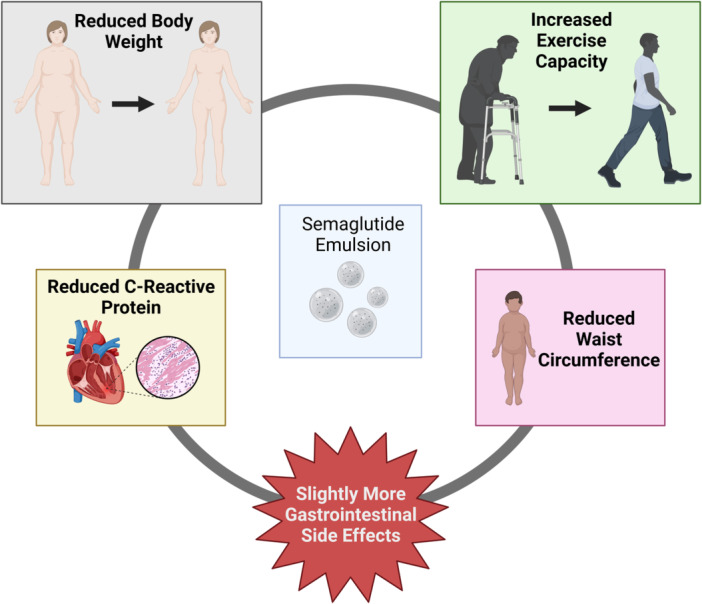

## 1 INTRODUCTION

Heart failure with preserved ejection fraction (HFpEF) is a prevalent and increasingly common form of heart failure in the United States, comprising more than half of all heart failure cases.^1^ This condition primarily affects individuals who are overweight or obese, raising intriguing questions about the intricate relationship between obesity and HFpEF.^2^ Recent research indicates that obesity might not merely coexist with HFpEF but could actively contribute to its development and progression.^3^

Individuals living with HFpEF and obesity face more adverse hemodynamic and clinical features. They experience a heavier symptom burden, reduced functional capacity, and a significantly impaired quality of life compared to HFpEF patients without obesity.^4^ This striking disparity underscores the urgent need for innovative treatment approaches tailored to this patient group.

One promising avenue of investigation involves pharmacotherapies that specifically target obesity. Among these, once‐weekly semaglutide, administered subcutaneously at a dose of 2.4 mg, has emerged as a potent glucagon‐like peptide 1 receptor agonist.^5^ It has received approval for long‐term weight management and has previously demonstrated its ability to induce substantial weight loss in individuals with overweight or obesity.^6^ Furthermore, it has exhibited favorable effects on cardiometabolic risk factors.

In light of these promising findings, a critical question arises: Can once‐weekly semaglutide at this dose level not only facilitate weight loss but also alleviate symptoms, reduce physical limitations, and enhance exercise capacity in patients afflicted by the challenging combination of HFpEF and obesity? To address this question, we embarked on a comprehensive study to assess the efficacy and safety of semaglutide in this specific patient population. This study aimed to shed light on whether this innovative pharmacotherapy can provide meaningful benefits beyond weight management for individuals grappling with the dual burden of HFpEF and obesity.

## 2 METHODS

### 2.1 Study design/patient selection

This was a retrospective cohort study. Retrospective studies involve the collection and analysis of historical data from electronic health records (EHRs) or other sources. The primary data source for this study was the EHR system of a large tertiary care hospital. The hospital's EHR system was chosen because it provided comprehensive patient information, including clinical notes, diagnostic tests, medication records, and demographics. Data were collected over a defined period, which typically spanned from January 1, 2022, to September 30, 2023. The selection of this time frame was critical to ensure that the data were relevant to the research question and that the patient population was representative of those with HFpEF and obesity who received Semaglutide during that period. To ensure ethical conduct, the trial was conducted following the principles outlined in the Declaration of Helsinki and under Good Clinical Practice guidelines. Our institute gave ethical review approval (Abbas Institute of Medical Sciences) after an ethics review board meeting (study ID # AIMS/23/52).

Participants were recruited based on specific eligibility criteria, including age (18 years or older), left ventricular ejection fraction (LVEF), body mass index (BMI), New York Heart Association (NYHA) functional class, 6‐min walk distance (6‐MWT), and the presence of certain cardiac findings indicative of HFpEF.

### 2.2 Data collection/outcomes

Access to the EHR system of a large tertiary care hospital, where the study was conducted, was essential. Authorized personnel, typically trained research assistants or healthcare professionals, were granted access to the EHR system. This access allowed them to retrieve patient data while adhering to strict data privacy and security protocols. Data extraction involved collecting a wide range of information, including demographic details (age, gender, etc.), comorbidities (e.g., diabetes, hypertension), medication history, vital signs (e.g., blood pressure, heart rate), laboratory results (e.g., HbA1c levels), and echocardiography reports (for assessing cardiac function). To ensure data accuracy and completeness, quality control measures were implemented. This included checking for missing or inconsistent data and resolving any discrepancies in the records. Data validation procedures were also employed to verify the accuracy of key data points. Different data sources within the EHR system were integrated to create a unified data set that allowed for comprehensive analysis. Data integration ensured that all relevant patient information was considered in the analysis. Before conducting statistical analyses, the data set underwent cleaning and preprocessing steps to address any outliers, missing values, or data anomalies. This step was crucial to ensure the integrity of the data used for analysis. The semaglutide treatment had been initiated at a lower dose and gradually escalated over 16 weeks to reach the maintenance dose of 2.4 mg. The primary endpoint of the original trial had been the percentage change in body weight from baseline to week 52. Additional secondary endpoints included the change in the 6‐MWT, a hierarchical composite endpoint measuring various clinical outcomes, and the change in C‐reactive protein (CRP) levels over the study period.

### 2.3 Data analysis

Initially, descriptive statistics were employed to summarize and present the baseline characteristics of the study population. For continuous variables (e.g., age, BMI, LVEF), means with standard deviations or medians with interquartile ranges were calculated, depending on the data distribution. Categorical variables, such as gender or comorbidities, were summarized using frequencies and percentages. To assess the impact of Semaglutide in patients with HFpEF and obesity, inferential statistical analyses were performed. Paired *t*‐tests are used to compare means of a continuous variable before and after an intervention and hi‐squared tests are employed to analyze categorical data. Linear regression analysis was used to assess the relationship between Semaglutide treatment and continuous outcomes while controlling for potential confounding factors. Logistic regression could have been used to evaluate binary outcomes to control for potential confounding variables and assess the independent effect of Semaglutide treatment, multivariate regression analysis was conducted. A two‐tailed p‐value of <0.05 was considered significant.

## 3 RESULTS

**Figure 1:**
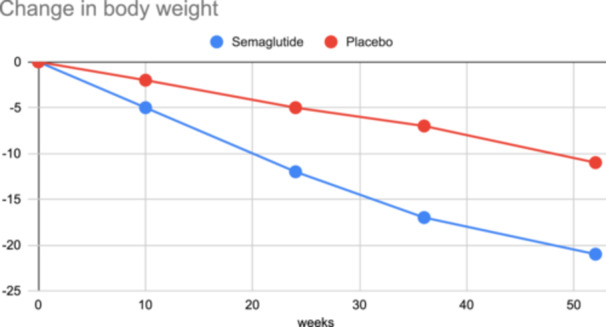
Changes from baseline to week 52 in the primary endpoint.

**Figure 2:**
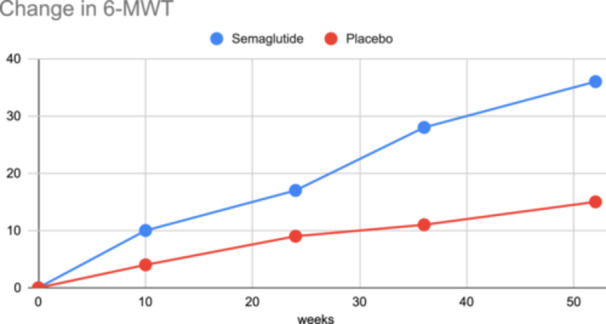
Changes from baseline to week 52 for confirmatory secondary endpoints.

The baseline characteristics of the study participants are summarized in Table 1. A total of 318 participants were enrolled, with 104 in the semaglutide group and 214 in the placebo group. The majority of participants were female (54.1%), and the median age was 69 years. The baseline characteristics, including body weight, BMI, waist circumference, systolic blood pressure, NT‐proBNP level, CRP level, and LVEF, were well‐balanced between the two groups. Coexisting conditions such as atrial fibrillation, hypertension, and coronary artery disease were also similar between the groups. The primary endpoint of the study was the percentage change in body weight from baseline to week 52 (Table 2). The results showed a significant reduction in body weight in the semaglutide group compared to the placebo group, with an estimated difference of −2.9% (95% CI −4.1% to −1.7%, *p*=0.001). This demonstrates the effectiveness of semaglutide in promoting weight loss in patients with HFpEF and obesity. Confirmatory secondary endpoints included changes in the 6‐min walk distance and CRP level from baseline to week 52. The semaglutide group exhibited a substantial improvement in the 6‐min walk distance, with an estimated difference of 15.1 meters (95% CI 5.8 to 24.4, *p*=0.002), indicating enhanced exercise capacity (Table 2). Additionally, there was a significant reduction in CRP levels in the semaglutide group compared to placebo, with an estimated difference of 0.47 (95% CI 0.32 to 0.62, *p*<0.001), suggesting an anti‐inflammatory effect. The hierarchical composite endpoint, which included various clinical and functional parameters, demonstrated a favorable outcome in the semaglutide group. The crude percentage of wins was 55.2% in the semaglutide group compared to 45.9% in the placebo group, with an estimated ratio of 1.20 (95% CI 0.92 to 1.56, *p*=0.187). Supportive secondary endpoints included changes in systolic blood pressure and waist circumference. Although the differences did not reach statistical significance, the semaglutide group showed a trend towards reduced systolic blood pressure (−1.8 mmHg) and decreased waist circumference (−6.7 cm). Furthermore, the study evaluated the percentage reduction in body weight at week 52 and found that a significantly higher proportion of participants in the semaglutide group achieved ≥10% reduction (61.5%, *p*=0.014), ≥15% reduction (49.2%, *p*=0.005), and ≥20% reduction (28.7%, *p*=0.010) compared to the placebo group. Exploratory endpoints included the percentage reduction in NT‐proBNP level, which showed a trend towards improvement in the semaglutide group (estimated ratio 0.80, 95% CI 0.56 to 1.14, *p*=0.240). Additionally, the analysis of adjudicated heart failure events using a time‐to‐event approach revealed a lower incidence in the semaglutide group, although it did not reach statistical significance (HR 0.23, 95% CI 0.05–1.05, *p*=0.075) (Table 3). In the analysis of adverse events, the Semaglutide group (7/104) exhibited a lower incidence of serious adverse events (6.7%) compared to the Placebo group (17/214) (7.9%). Moreover, serious adverse events leading to discontinuation were more frequent in the Semaglutide group (14.3%) compared to the Placebo group (11.8%). Gastrointestinal disorders were reported in both groups, with a slightly higher occurrence in the Semaglutide group (14.3%) compared to the Placebo group (5.9%). Adverse events leading to discontinuation were more prevalent in the Semaglutide group (28.6%) compared to the Placebo group (5.9%), with gastrointestinal disorders being a notable contributor. Fatal events were observed in both groups, with a higher incidence in the Semaglutide group (42.9%) compared to the Placebo group (29.4%). Among the most frequent serious adverse events, cardiac disorders were reported in both groups, albeit with a slightly higher frequency in the Placebo group (23.5% vs. 14.3% in Semaglutide). Atrial fibrillation and cardiac failure were more prevalent in the Placebo group (11.8%) compared to the Semaglutide group. Infection or infestation and gastrointestinal disorders were reported with similar frequencies in both groups. Figure 1 shows changes from baseline to week 52 in the primary endpoint and Figure 2 demonstrates changes from baseline to week 52 for confirmatory secondary endpoints.

**Table 1:** Baseline characteristics.

Characteristic	Semaglutide (N=104)	Placebo (N=214)	Total (N=318)
Female sex—no. (%)	59 (56.7)	113 (52.8)	172 (54.1)
Median age (IQR)—yr	70 (62–75)	69 (63–75)	69 (63–75)
Ethnic group—no. (%)			
Punjabi	3 (2.9)	8 (3.7)	11 (3.5)
Kashmiri	97 (93.3)	204 (95.3)	301 (94.7)
Other	0 (0.0)	2 (0.9)	2 (0.6)
Median body weight (IQR)—kg	104.7 (92.4–120.1)	105.3 (92.4–122.0)	105.1 (92.4–120.8)
Median BMI (IQR)	37.2 (33.9–41.1)	36.9 (33.3–41.6)	37.0 (33.7–41.4)
BMI stratum—no. (%)			
30 to <35	35 (33.7)	73 (34.1)	108 (33.9)
≥35	69 (66.3)	141 (65.9)	210 (66.1)
Median waist circumference (IQR)—cm	119.0 (110.5–127.1)	120.0 (110.5–129.0)	119.4 (110.5–128.0)
Median systolic blood pressure (IQR)—mm Hg	133 (122–145)	132 (120–142)	133 (121–144)
Median NT‐proBNP level (IQR)—pg/ml	414.4 (229.2–1014.0)	499.8 (204.7–1025.0)	450.8 (218.2–1015.0)
Median CRP level (IQR)—mg/liter	3.8 (1.9–7.0)	3.9 (2.0–8.4)	3.8 (1.9–7.7)
Median LVEF (IQR)—%	57.0 (50.0–60.0)	57.0 (50.0–60.0)	57.0 (50.0–60.0)
LVEF stratum—no. (%)			
45 to <50%	14 (13.5)	26 (12.1)	40 (12.6)
50% to 59%	47 (45.2)	92 (43.0)	139 (43.7)
≥60%	43 (41.3)	96 (44.9)	139 (43.7)
Median 6‐min walk distance (IQR)—m	316.0 (251.0–386.0)	325.8 (232.4–392.0)	320.0 (240.0–389.0)
Hospitalization for heart failure within 1 year—no. (%)	16 (15.4)	34 (15.9)	50 (15.7)
Coexisting conditions at screening—no. (%)			
Atrial fibrillation	54 (51.9)	102 (47.7)	156 (49.1)
Hypertension	82 (78.8)	163 (76.2)	245 (77.0)
Coronary artery disease	23 (22.1)	47 (22.0)	70 (22.0)
NYHA functional class—no. (%)			
II	69 (66.3)	125 (58.4)	194 (61.0)
III or IV	35 (33.7)	89 (41.6)	124 (39.0)
Concomitant medication—no. (%)			
Diuretic	82 (78.8)	161 (75.2)	243 (76.4)
Loop diuretic	60 (57.7)	116 (54.2)	176 (55.3)
Thiazide	22 (21.2)	42 (19.6)	64 (20.1)
MRA	30 (28.8)	60 (28.0)	90 (28.3)
ACEI, ARB, or ARNI	77 (74.0)	150 (70.1)	227 (71.4)
Beta‐blocker	80 (76.9)	156 (72.9)	236 (74.2)
SGLT2 inhibitor	5 (4.8)	11 (5.1)	16 (5.0)

**Table 2:** Efficacy endpoint.

Endpoint	Semaglutide (N=104)	Placebo (N=214)	Estimated difference or ratio (95% CI)	P value
Primary end point				
Percentage change in body weight from baseline to week 52	−10.7	−7.8	−2.9 (−4.1–−1.7)	0.001
Confirmatory secondary end points				
Change from baseline to week 52 in 6‐min walk distance—m	18.7	3.6	15.1 (5.8–24.4)	0.002
Change from baseline to week 52 in CRP level—%	−37.2	−12.1	0.47 (0.32–0.62)	<0.001
Hierarchical composite end point—crude percentage of wins	55.2	45.9	1.20 (0.92–1.56)	0.187
Supportive secondary end points				
Change from baseline to week 52 in systolic blood pressure—mm Hg	−6.2	−4.4	−1.8 (−3.7–0.1)	0.065
Change from baseline to week 52 in waist circumference—cm	−9.8	−3.1	−6.7 (−8.9–−4.5)	0.001
Percentage reduction in body weight at week 52—% of participants	≥10% reduction	61.5	10.0 (2.1–17.9)	0.014
	≥15% reduction	49.2	29.7 (8.7–50.7)	0.005
	≥20% reduction	28.7	50.5 (13.3–87.6)	0.010
Attainment of anchor‐based threshold for change in 6‐min walk distance—% of participants	38.0	34.6	1.10 (0.72–1.69)	0.671
Exploratory end points assessed in the overall population				
Percentage reduction from baseline to week 52 in NT‐proBNP level	−15.8	−6.7	0.80 (0.56–1.14)	0.240
Adjudicated heart failure event (hospitalization or urgent visit for heart failure), time‐to‐event analysis—no. of events	2	9	0.23 (0.05–1.05)	0.075

**Table 3:** Adverse events reported.

Adverse Event	Semaglutide group (7/104)	Placebo group (17/214)
Serious adverse events	3 (42.9%)	8 (47.1%)
Serious adverse events leading to discontinuation	1 (14.3%)	2 (11.8%)
Gastrointestinal disorder	1 (14.3%)	1 (5.9%)
Adverse events leading to discontinuation	2 (28.6%)	1 (5.9%)
Gastrointestinal disorder leading to discontinuation	1 (14.3%)	0 (0.0%)
Fatal event	3 (42.9%)	5 (29.4%)
Most frequent serious adverse events		
‐ Cardiac disorder	1 (14.3%)	4 (23.5%)
‐ Atrial fibrillation	0 (0.0%)	2 (11.8%)
‐ Cardiac failure	0 (0.0%)	2 (11.8%)
‐ Infection or infestation	1 (14.3%)	4 (23.5%)
‐ Gastrointestinal disorder	1 (14.3%)	1 (5.9%)
‐ Nervous system disorder	1 (14.3%)	1 (5.9%)
‐ Renal or urinary disorder	1 (14.3%)	1 (5.9%)
‐ Respiratory, Thoracic, or Mediastinal event	0 (0.0%)	2 (11.8%)
‐ Musculoskeletal or connective‐tissue event	1 (14.3%)	1 (5.9%)
‐ Injury, poisoning, or procedural event	1 (14.3%)	1 (5.9%)
‐ Metabolism or nutrition disorder	0 (0.0%)	1 (5.9%)
‐ Hepatobiliary disorder	0 (0.0%)	0 (0.0%)
‐ General disorder or administration‐site reaction	0 (0.0%)	1 (5.9%)

## 4 DISCUSSION

In this retrospective study, involving patients with HFpEF and obesity, once‐weekly semaglutide at a dose of 2.4 mg led to reductions in HF‐related symptoms and physical limitations (measured in 6‐MWT) and a greater degree of weight loss than placebo at 52 weeks. Furthermore, the analysis showed that semaglutide outperformed the placebo in terms of achieving more favorable outcomes in the hierarchical composite endpoint evaluation. Additionally, semaglutide treatment resulted in a more significant reduction in CRP levels compared to the placebo. Notably, individuals receiving semaglutide experienced fewer serious adverse events when compared to those on the placebo. Moreover, the rate of discontinuation due to serious adverse events was similar between the semaglutide and placebo groups.

Patients diagnosed with HFpEF often experience a significant burden of symptoms and physical limitations, which severely impacts their quality of life.^7^, ^8^, ^9^ These symptoms and functional impairments are considered critical aspects of managing the condition, alongside efforts to prevent hospitalizations and mortality. Patient feedback has revealed that individuals with HFpEF place a high value on symptom reduction and improved physical function, sometimes even on par with the importance of avoiding death.^10^, ^11^, ^12^ Regulatory bodies like the Food and Drug Administration (FDA) have recognized the significance of these factors, suggesting that a treatment for heart failure could be considered based solely on improvements in symptoms and physical function.^13^ Unfortunately, there has been a lack of effective treatments targeting these crucial outcomes in HFpEF patients, highlighting a substantial unmet medical need.

Moreover, while HFpEF is often underdiagnosed, especially in individuals with obesity, epidemiological data reveals that a majority of HFpEF patients indeed have obesity.^14^, ^15^ Emerging evidence suggests that adipose tissue may play a pivotal role in the development, progression, and adverse consequences of HFpEF.^15^ Specifically, visceral adiposity is linked to increased inflammation, left ventricular hypertrophy, insulin resistance, and various cardiac dysfunctions, including diastolic and systolic left ventricular dysfunction.^16^ Additionally, it contributes to arterial, skeletal muscle, and physical impairments. Among HFpEF patients, those with the obesity phenotype exhibit distinct clinical and hemodynamic characteristics, such as expanded plasma volume, reduced venous capacitance, elevated exercise pulmonary wedge pressures, adverse hemodynamic response to diuresis, higher inflammatory markers, pronounced hypertension, severe symptoms, and exercise intolerance.^17^, ^18^ Obesity also leads to natriuretic peptide deficiency due to reduced production and increased clearance, resulting in decreased vasodilation and natriuresis capacity.^19^

Despite the strong association between obesity, excess adiposity, and worse HFpEF outcomes, and despite previous evidence suggesting that lifestyle modifications and weight loss can improve health status and exercise capacity in HFpEF patients with obesity, there has been a notable absence of prospective trials investigating pharmacological treatments specifically tailored to address the obesity‐related phenotype of HFpEF.^6^, ^7^ This gap underscores the need for novel and effective therapeutic approaches in this patient population.

The substantial improvement in the 6‐min walk distance observed in our trial holds significant clinical relevance. Even in cases where patients have well‐managed HFpEF and are in stable condition, their objectively measured physical function remains markedly impaired. Impaired physical function serves as an independent predictor of various adverse outcomes, including reduced quality of life, increased hospitalizations, loss of independence, nursing home placement, and mortality.^20^ Historically, most clinical trials assessing different medications for HFpEF have yielded neutral results when it comes to exercise‐related outcomes like the 6‐min walk distance or cardiopulmonary exercise testing. Notably, the absolute increase in the 6‐min walk distance observed in our semaglutide trial is quite remarkable. It surpasses the improvements seen in the HF‐ACTION trial, which focused on exercise training for heart failure with reduced ejection fraction, and is similar in magnitude to those observed in exercise training trials for HFpEF patients.^21^ In summary, our findings suggest that semaglutide could offer a valuable therapeutic approach to managing HFpEF in patients with obesity.

Furthermore, our study sheds light on a long‐standing debate surrounding weight and weight loss in individuals with heart failure. Past observational reports indicated that a higher BMI might be associated with a better prognosis in heart failure patients, leading to the concept of the “obesity paradox.”^22^ However, these observations didn't distinguish between unintentional weight loss (often related to poor prognosis) and intentional weight loss resulting from various interventions. Small observational studies and a randomized trial focusing on intentional weight loss in heart failure patients with obesity indicated associations with reduced symptom severity, improved functional status, and enhanced quality of life.^23^ Our data extend these findings and show that weight loss with semaglutide (at a 2.4 mg dose) is a beneficial strategy for patients with HFpEF and obesity. Future trials could explore whether similar benefits are achievable with different weight loss interventions or in other populations, such as those with heart failure and reduced ejection fraction and obesity.

Several potential mechanisms may underlie the treatment benefits associated with semaglutide in this patient group. The observed trajectory of symptom reduction and physical improvement, coupled with weight loss and decreased visceral adipose tissue, suggests that weight loss likely plays a pivotal role in these benefits. Semaglutide also led to significant reductions in CRP levels, systolic blood pressure, and NT‐proBNP levels, indicating potential favorable anti‐inflammatory and hemodynamic effects. It remains unclear whether these benefits are solely attributable to weight loss, other direct mechanisms, or a combination of factors. Despite the common association of higher BMIs with lower NT‐proBNP levels, we observed a substantial reduction in NT‐proBNP levels with semaglutide, even alongside significant weight loss.^24^ Additionally, fewer adjudicated heart failure events occurred in the semaglutide group, further suggesting substantial decongestive and favorable hemodynamic effects. This aligns with the lower number of reported cardiovascular serious adverse events with semaglutide, including events like atrial fibrillation and flutter, which are expected to decline with improvements in hemodynamic status and reduced inflammation. Together, these findings support the notion that the benefits of semaglutide encompass more than just weight loss; they potentially signify concurrent improvements in the pathophysiological processes underpinning HFpEF itself.

This study's findings present significant insights into the management of HFpEF, particularly in patients with obesity. However, it is important to acknowledge certain limitations that warrant consideration. Firstly, the study primarily focused on a specific subgroup of HFpEF patients—those with obesity. As such, the generalizability of these results to broader HFpEF populations, such as nonobese patients or those with heart failure with reduced ejection fraction (HFrEF), may be somewhat limited. The unique characteristics and responses observed in obese HFpEF patients may not directly translate to other subsets of heart failure patients.

Secondly, the study's follow‐up duration was limited to 52 weeks. While this timeframe allowed for the observation of significant improvements in various endpoints, including symptoms, exercise capacity, and inflammatory markers, the long‐term effects of semaglutide treatment beyond 1 year remain uncertain. It is crucial to assess the durability and sustained benefits of this pharmacologic intervention over more extended periods, especially given the chronic nature of HFpEF. Additionally, the study primarily investigated the effects of semaglutide, with a specific focus on weight loss as a potential mechanism for symptom improvement. Although the results indicated substantial reductions in symptoms and physical limitations that correlated with weight loss, the study did not comprehensively explore the mechanisms underlying these benefits. Future research should delve deeper into the specific pathways through which semaglutide exerts its effects, examining whether factors beyond weight loss, such as anti‐inflammatory or hemodynamic mechanisms, play significant roles. Lastly, while the study demonstrated a reduction in serious adverse events with semaglutide treatment, it is crucial to acknowledge that clinical trials inherently have a controlled environment, and real‐world scenarios may differ. Continuous monitoring of safety and adverse events in broader patient populations receiving semaglutide will be essential to establish its long‐term safety profile.

## 5 CONCLUSION

In individuals diagnosed with HFpEF and coexisting obesity, the administration of once‐weekly semaglutide at a dosage of 2.4 mg resulted in notable improvements. This treatment led to substantial reductions in symptoms associated with heart failure, as well as a marked enhancement in physical function. Additionally, the semaglutide group exhibited more significant weight loss when compared to the placebo group. These findings collectively suggest that semaglutide has the potential to offer meaningful benefits to patients grappling with HFpEF and obesity.

## AUTHOR CONTRIBUTIONS

**Ayesha Rehman**, **Shahab Saidullah**, **Muhammad Asad** did concept and methodology. **Jahanzeb Malik** and **Muhammad F. Khan** supervised and wrote final draft. **Jahanzeb Malik**, **Umer R. Gondal**, **Amna Ashraf**, and **Muhammad F. Khan** wrote first draft, literature search. **Waheed Akhtar** collected data from his institute and supervised data anonymization. **Amin Mehmoodi** supervision, concept, wrote first draft, and finalized it.

## CONFLICT OF INTEREST STATEMENT

The authors declare no conflict of interest

## Data Availability

The data that support the findings of this study are available on request from the corresponding author. The data are not publicly available due to privacy or ethical restrictions.
